# Supplementary material for: Orthogonal luminescence lifetime encoding by intermetallic energy transfer in heterometallic rare-earth MOFs
Source: Nat Commun. 2023 Feb 22;14:981. doi: 10.1038/s41467-023-36576-z (PMC9947006; doi:10.1038/s41467-023-36576-z)
Supplement: Supplementary file 1 — Supplementary Information [file 41467_2023_36576_MOESM1_ESM.docx]

Supporting Information

Multiplexed luminescence lifetime encoding by intermetallic energy transfer in rare-earth MOFs

Jacob I. Deneff, Lauren E.S. Rohwer, Kimberly S. Butler, Bryan Kaehr, Dayton J. Vogel, Ting S. Luk, Raphael A. Reyes, James E. Martin, Dorina F. Sava Gallis*

**Supplementary Table 1.** Crystal data and structure refinement for compound **1**.

Empirical formula C204 Eu18 O93.26

Formula weight 6677.48

Temperature 116 K

Wavelength 1.54178 Å

Crystal system, space group Hexagonal P 6_3_/m m c

Unit cell dimensions a = 22.0812(3) Å

b = 22.0812(3) Å

c = 25.2784(7) Å

Volume 10674.0(4) Å^3^

Z, Calculated density 1, 1.039 g/cm^3^

F(000) 3104.0

Crystal size 0.34 x 0.18 x 0.12 mm

Theta range for data collection 2.310 to 55.970°

Reflections collected / unique 2600/2377

R indices R1 = 0.1353, wR2 = 0.3167

Largest diff. peak and hole -4.752 to 6.088 eÅ^-3^

The general chemical formula for the compounds presented herein is:

[(M9) (μ3-OH)12 (μ3-O)2 (H2O)9(TCPB)3]- [NH2(CH3)2]+ ‧12(DMF).

In monometallic compositions, M= Eu, Yb, or Gd (compounds **1**, **4**, **7**). For dimetallic and trimetallic compositions (compounds **2**, **3**, **5**, **6**, **8**, **9**, **10**, **11**, **12**, **13**) the metal distribution is a fractional combination of those three metals based on each individual composition, as outlined in **Supplementary Table 1**. Details regarding the data collection parameters are summarized in **Supplementary Table 1**, and ORTEP drawing of compound **1** is depicted in **Supplementary Figure 1**. There are A- and B-level alerts for the crystallographic data. These are due to several correlated reasons. First, the data has a low resolution; the crystal was weakly diffracting, which is a common and well documented issue with highly porous, low density metal-organic framework materials that contain voids. Second, the structure is disordered; two positions are modeled, but there is some remaining electron density.


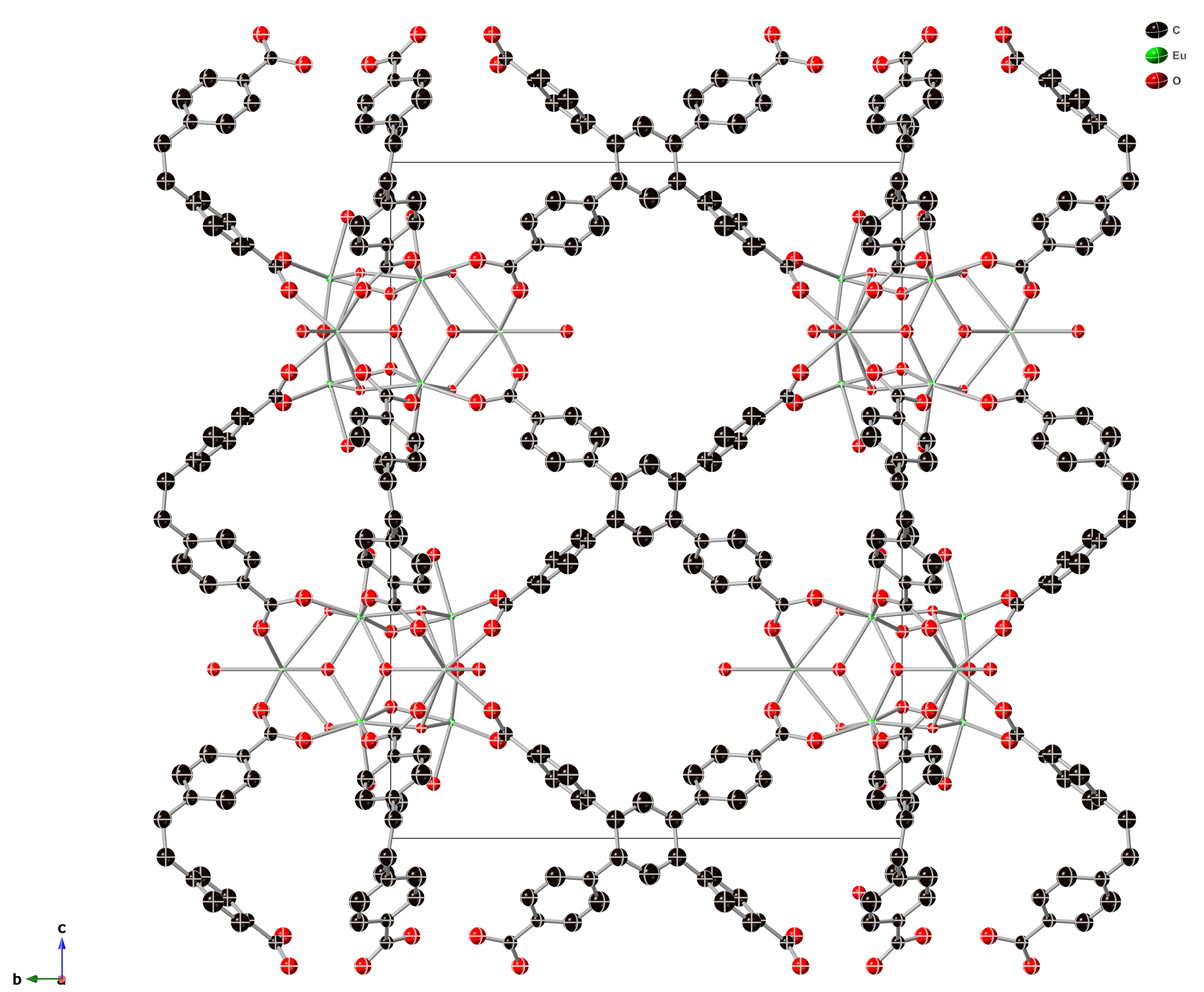


**Supplementary Figure 1.** ORTEP-style illustration for compound **1**. Thermal ellipsoids are shown at the 50% probability level, and hydrogen atoms are omitted for clarity.


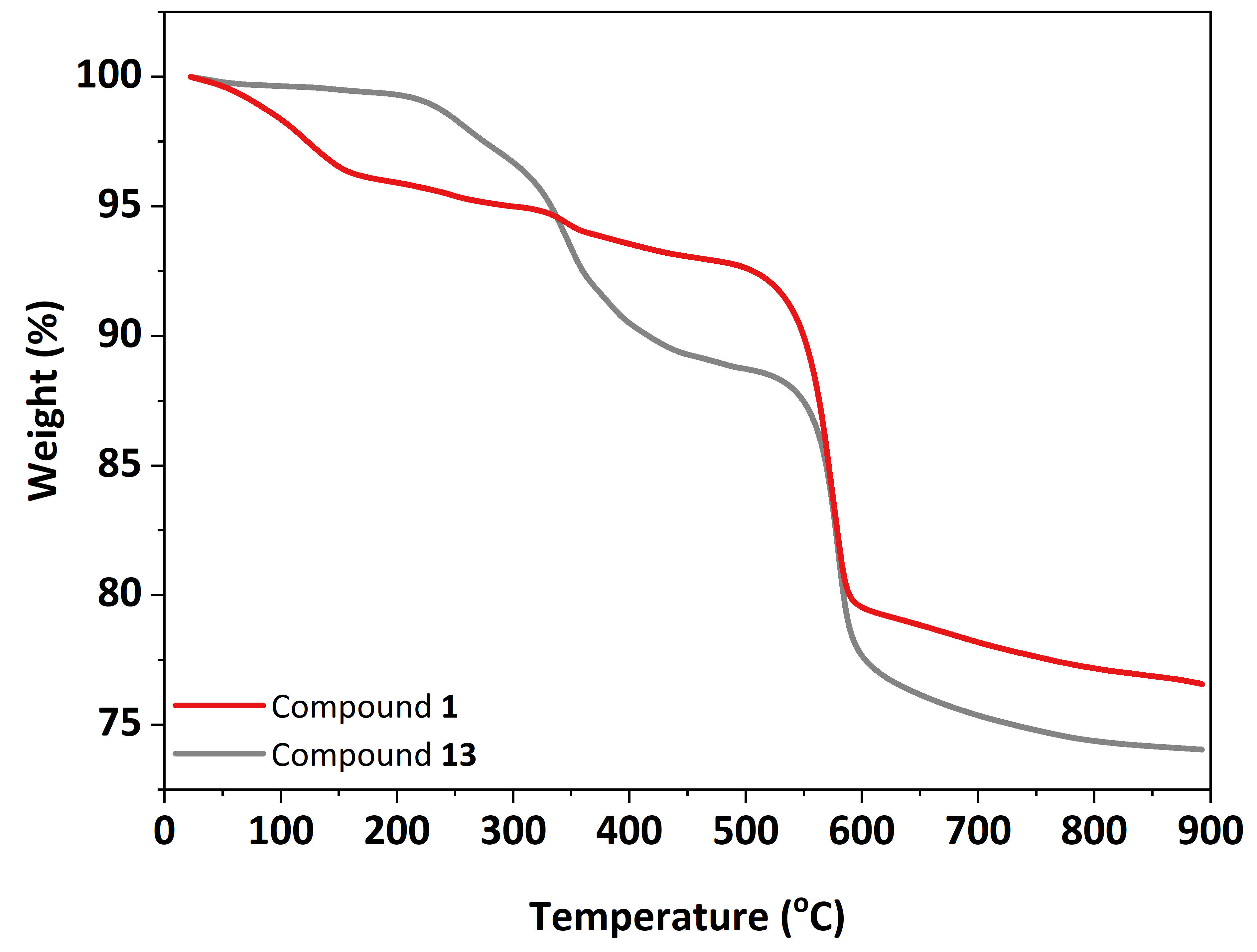


**Supplementary Figure 2.** Thermogravimetric analyses (TGA) for two representative compounds, compounds **1** and **13**, highlighting the high thermal stability intrinsic to these materials.


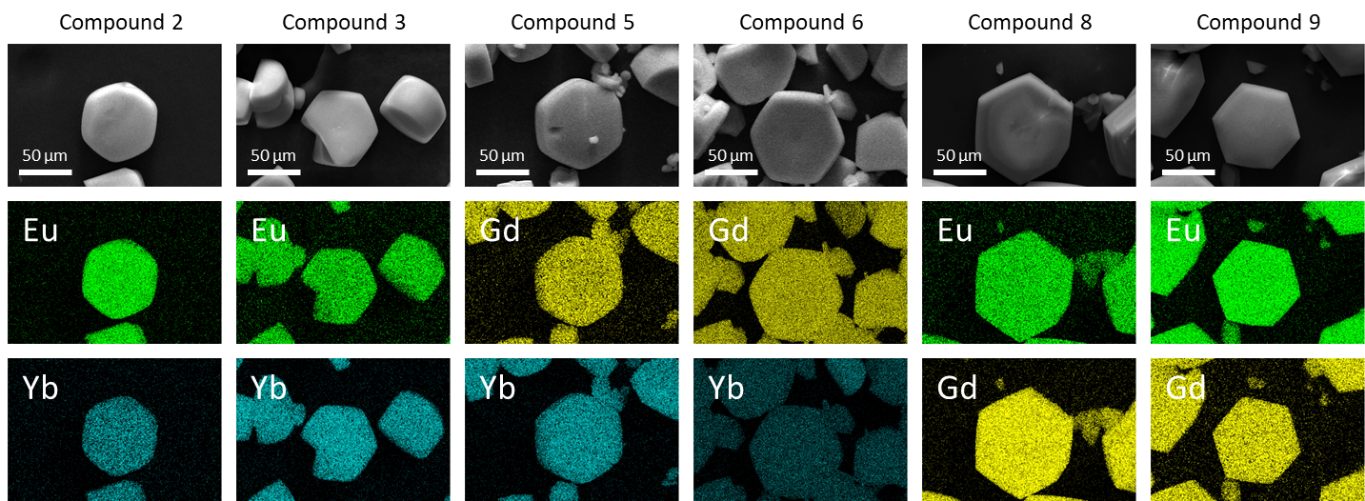


**Supplementary Figure 3.** SEM images of dimetallic compounds **2**, **3**, **5**, **6**, **8** and **9** highlighting homogeneous distribution of all metals at the single crystal level.


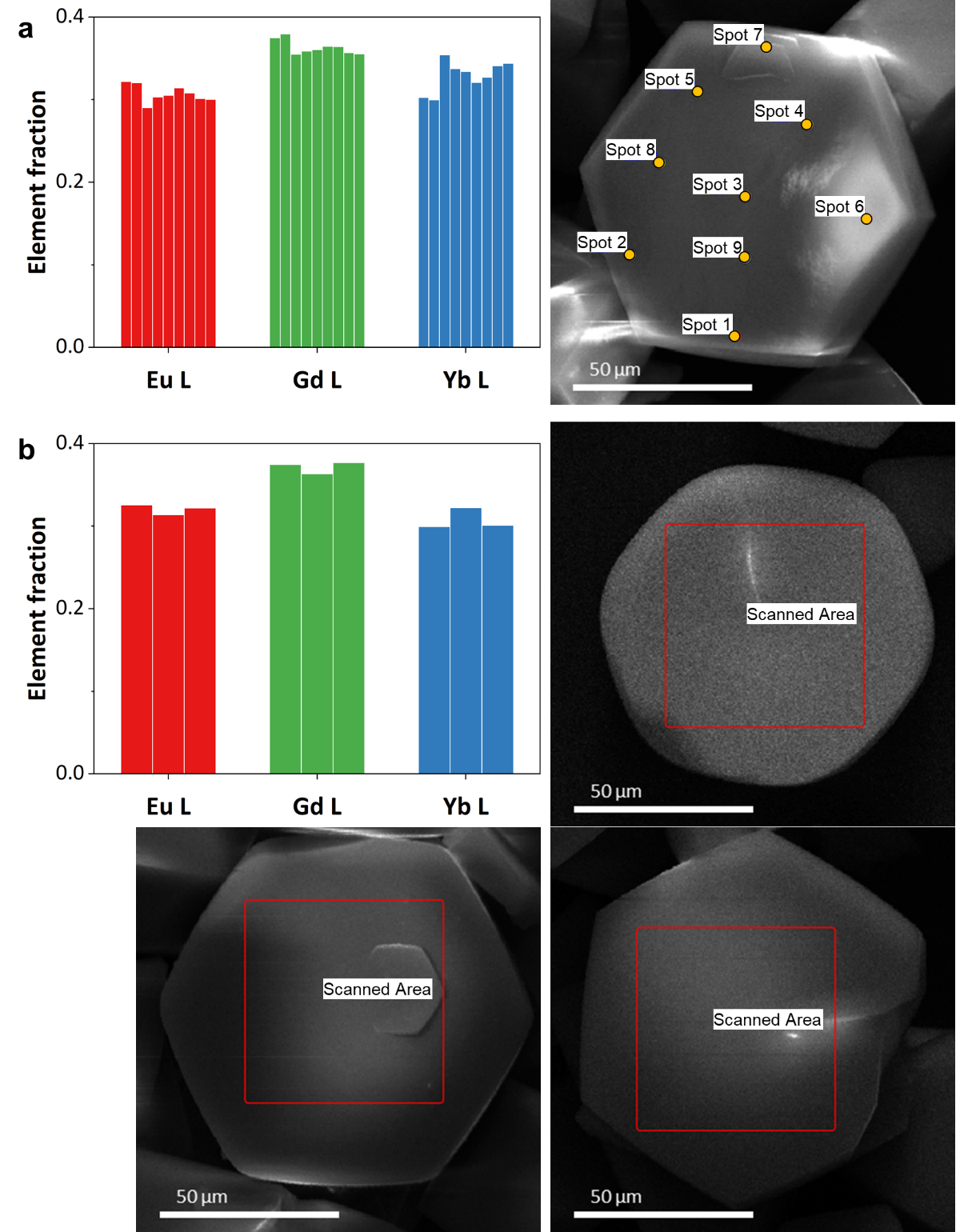


**Supplementary Figure 4**. Histograms showing the proportion of elements in compound **13**. a. Nine separate points on a single crystal are used to show homogeneous distribution of metals within individual crystals; b. area scans of three different crystals are used to show homogeneous composition in the bulk material. The images below the histograms show the points and the areas the data was taken from.


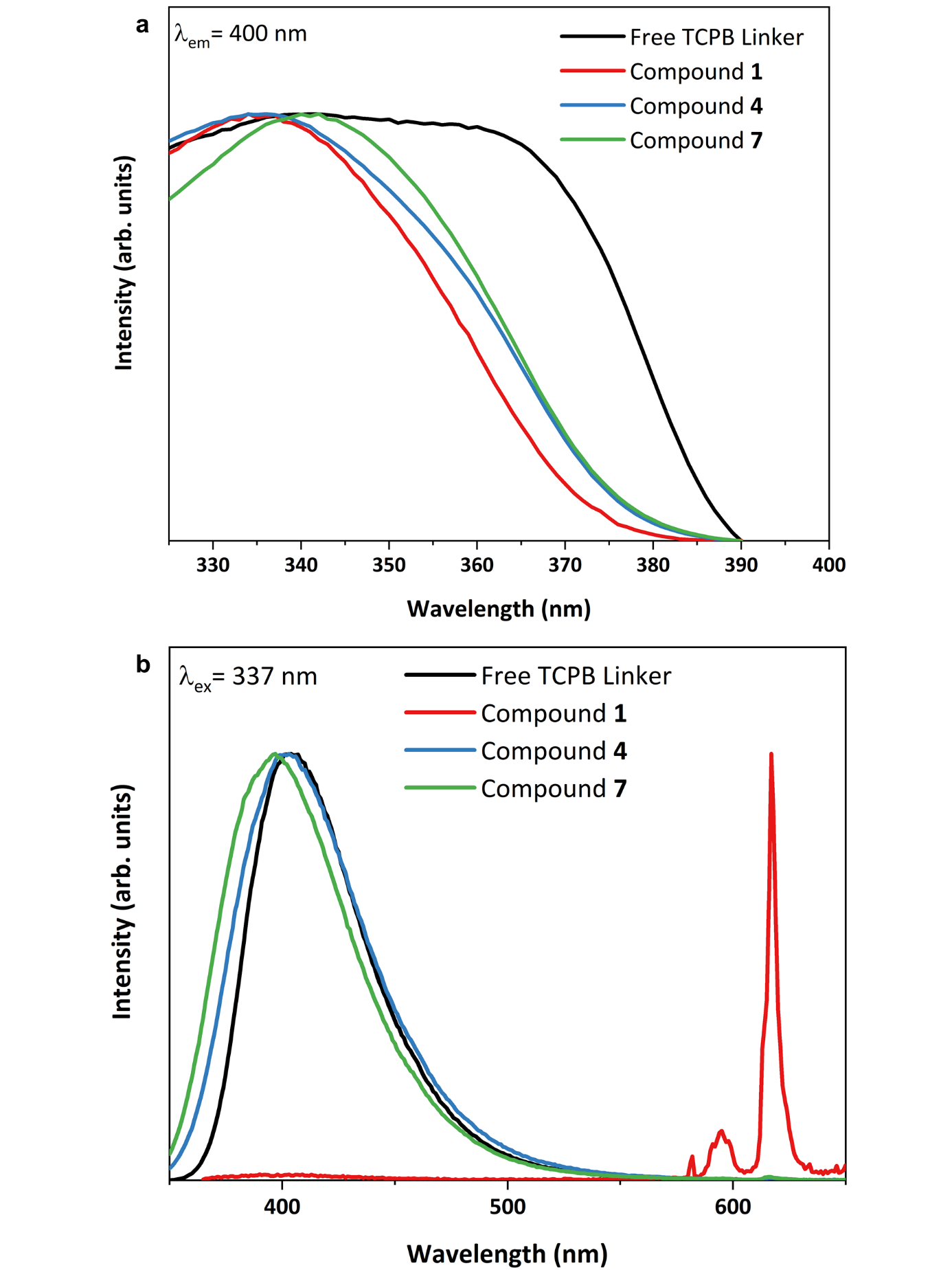


**Supplementary Figure 5**. a. Photoluminescent excitation spectra for the TCPB linker and compounds **1**, **4** and **7**, measured at 400 nm to monitor linker excitation; b. corresponding photoluminescent emission spectra for the TCPB linker and each of the single metal compounds, compounds **1**, **4** and **7**.

**
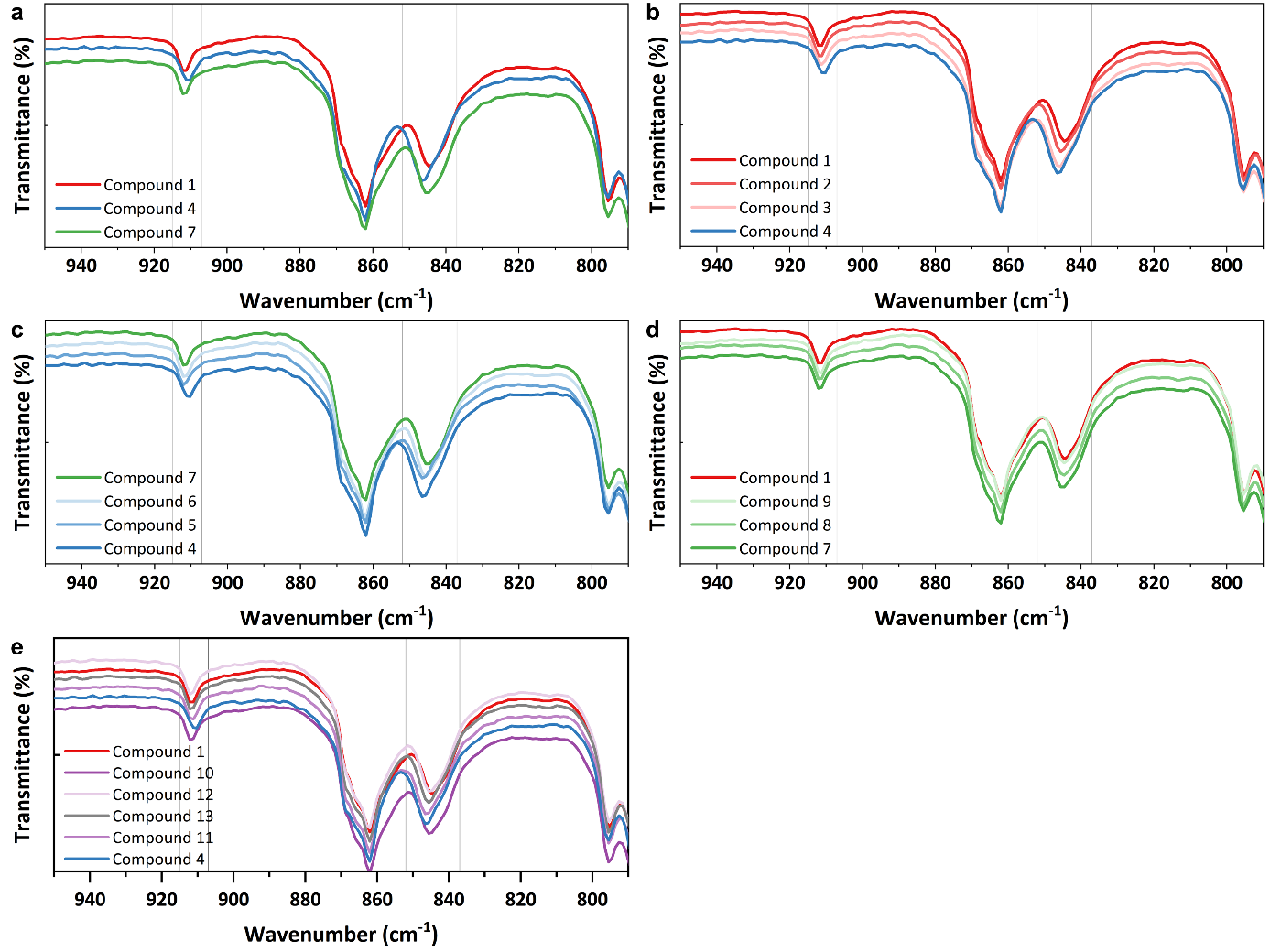
**

**Supplementary Figure 6.** IR spectra of all compounds illustrating the effect of composition on peak location for OH groups within the clusters. a. Homometallic compositions, b.-d. Dimetallic compositions, e. Trimetallic compositions. In each plot traces are ordered according to Yb content from lowest (top) to highest (bottom).


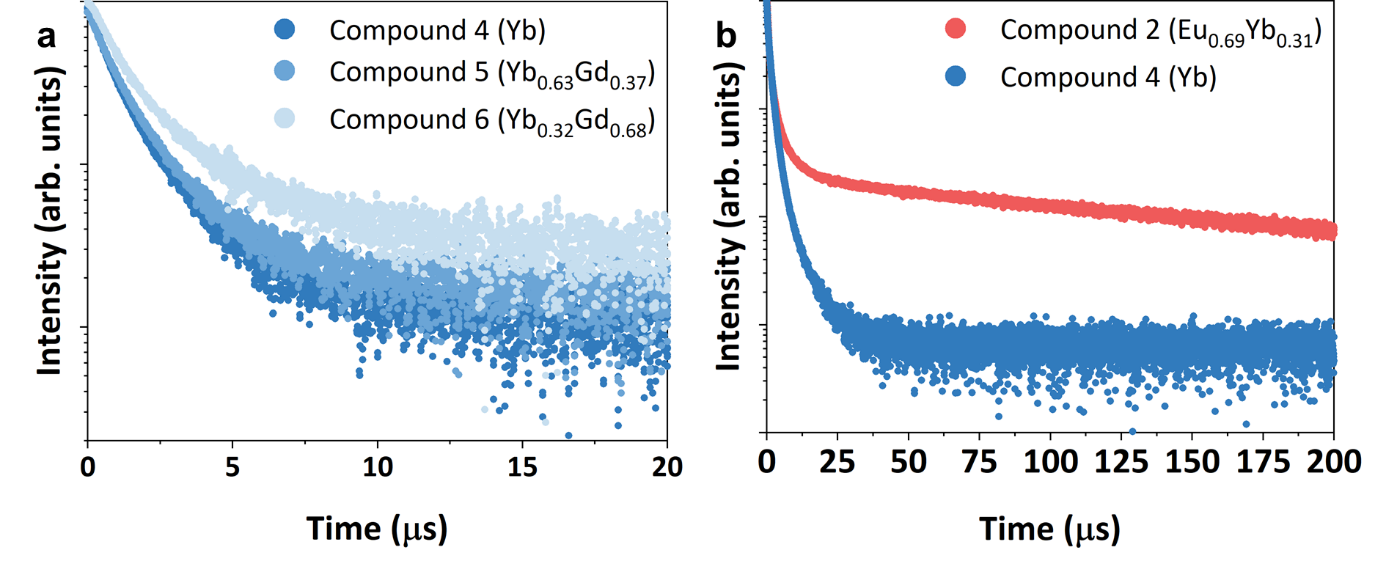


**Supplementary Figure 7.** a. NIR decay curves for compounds containing Yb and Gd; b. NIR decay curves comparing Yb decay with and without Eu, showing the long-lived emission of compound **2** caused by internal energy transfer.


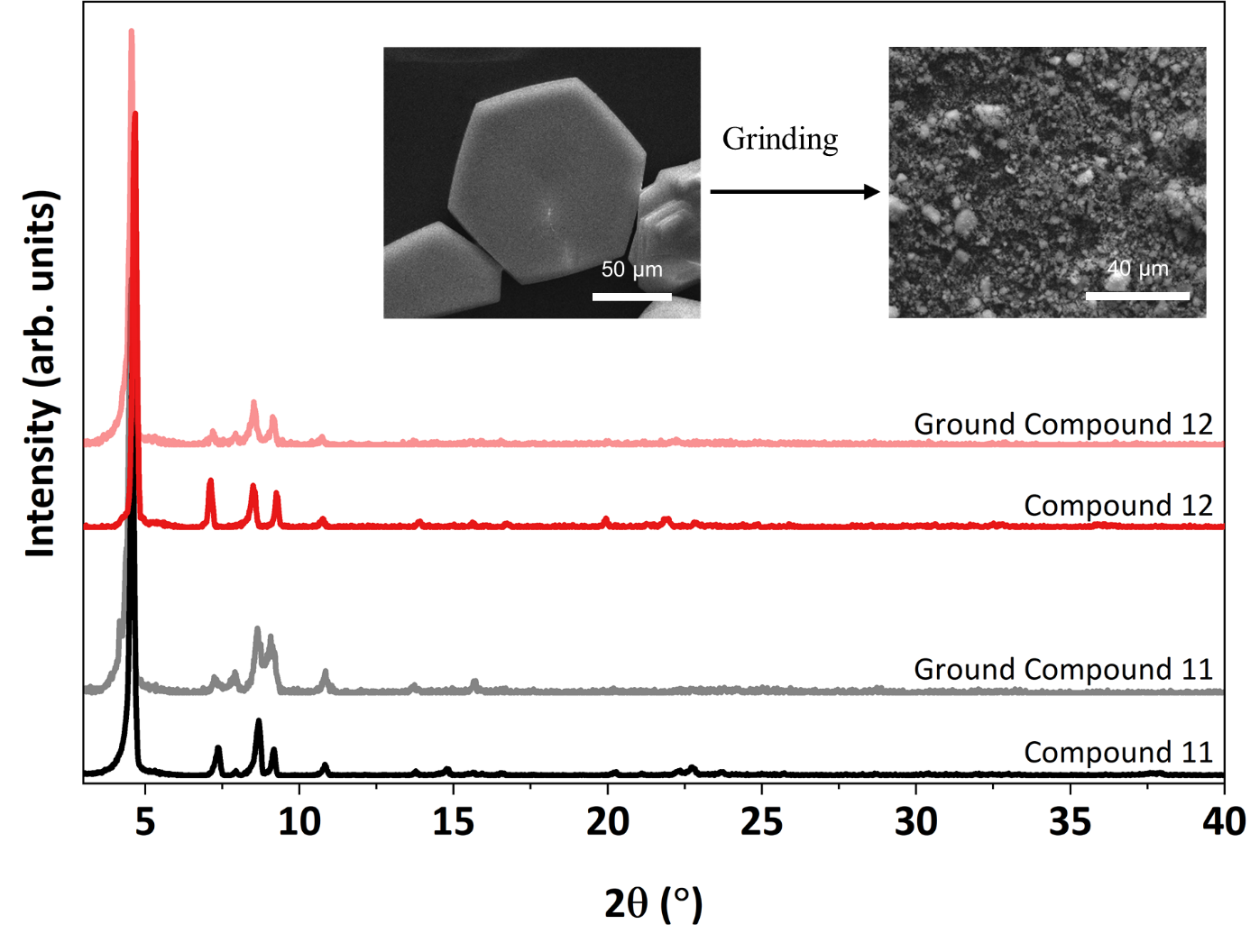


**Supplementary Figure 8.** Comparison of the PXRD patterns for as-made and ground compounds **11** and **12**, showing no impact on the crystallinity of the compounds after grinding.


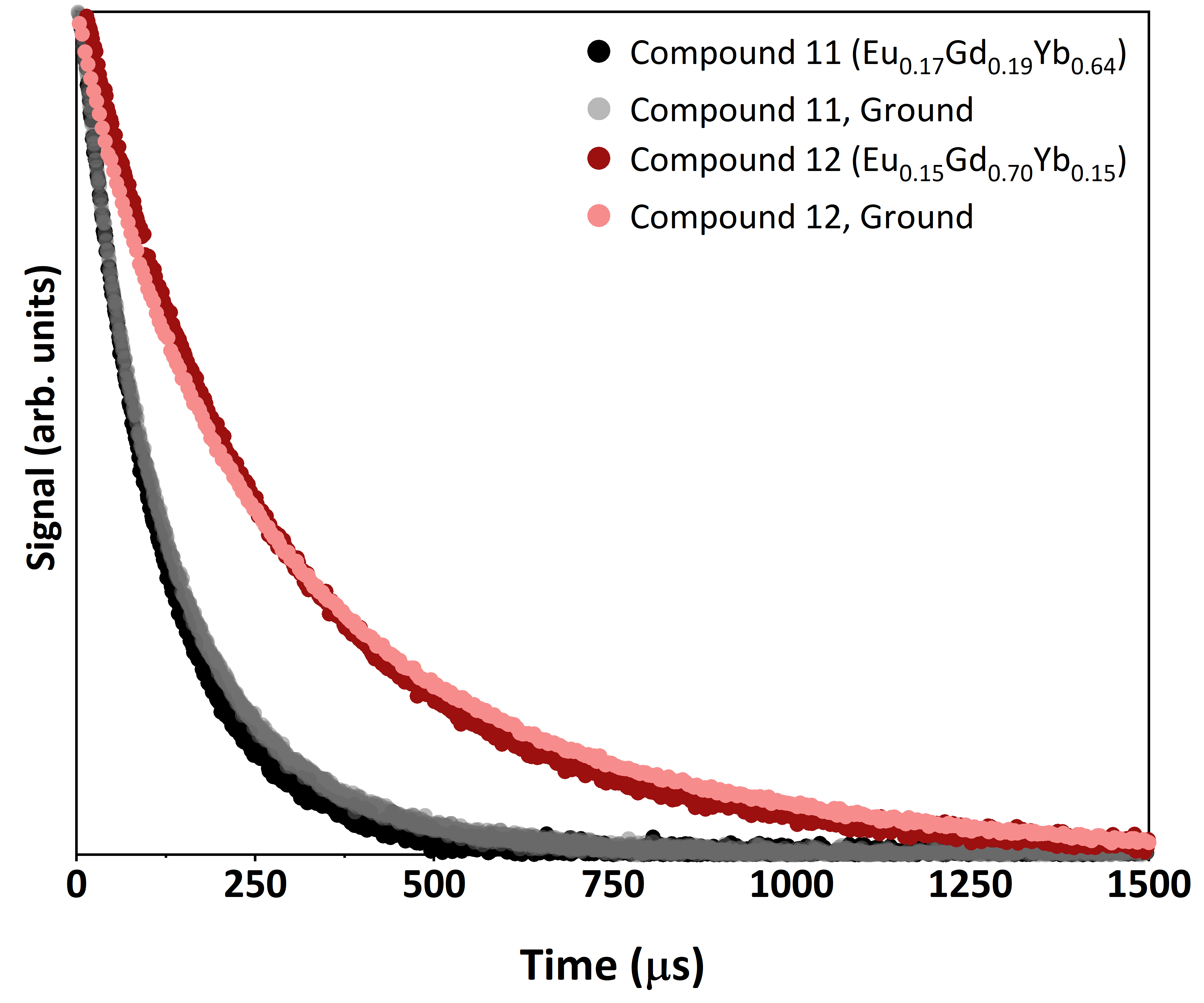


**Supplementary Figure 9**. Comparison of luminescence decay for as-made and ground compounds **11** and **12** in the visible range, showing little to no change occurs in the lifetime of the compounds after grinding. λ_ex_ = 337 nm


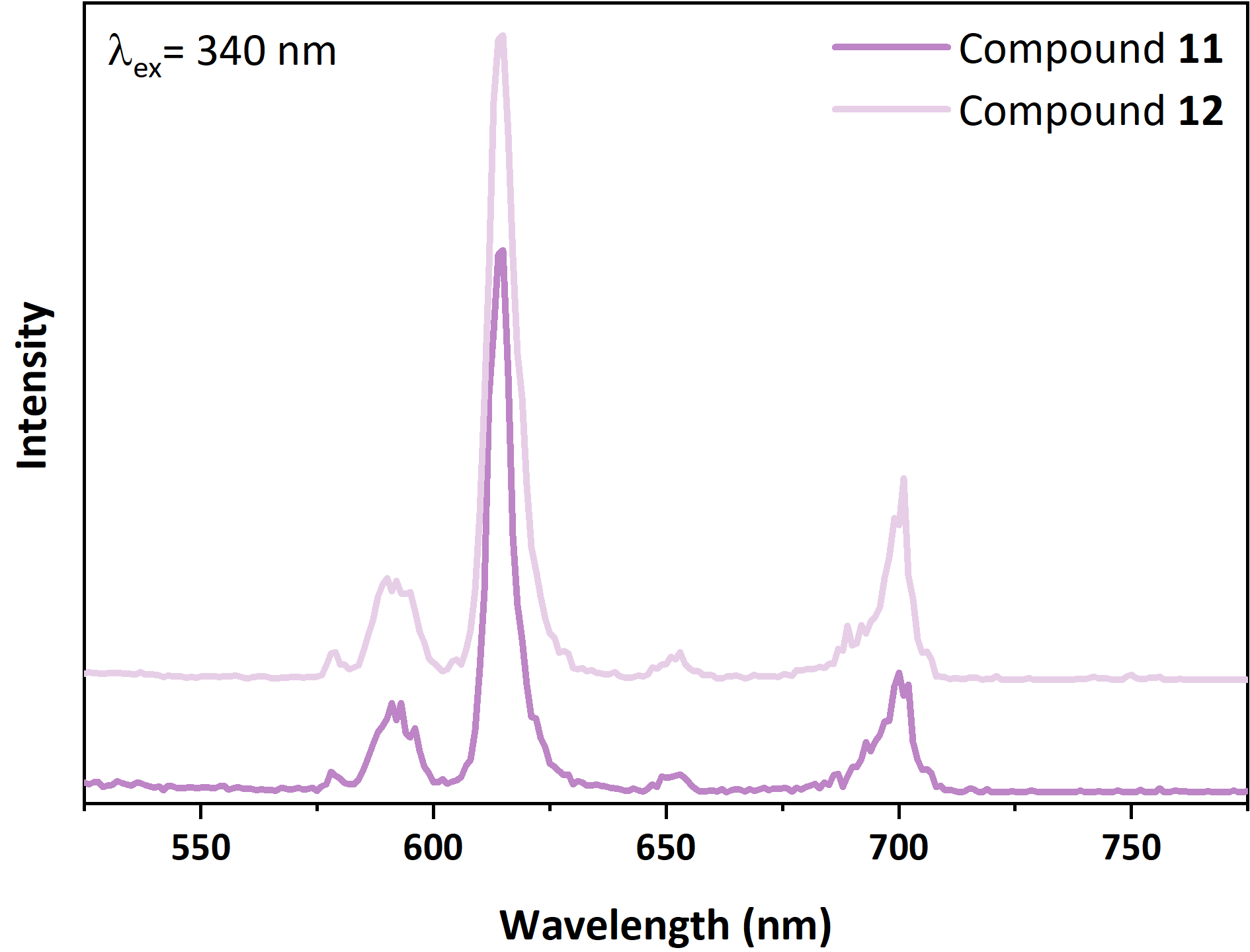


**Supplementary Figure 10.** Photoluminescent spectra of compounds **11** and **12** taken on the plate reader, demonstrating their identical emissions.


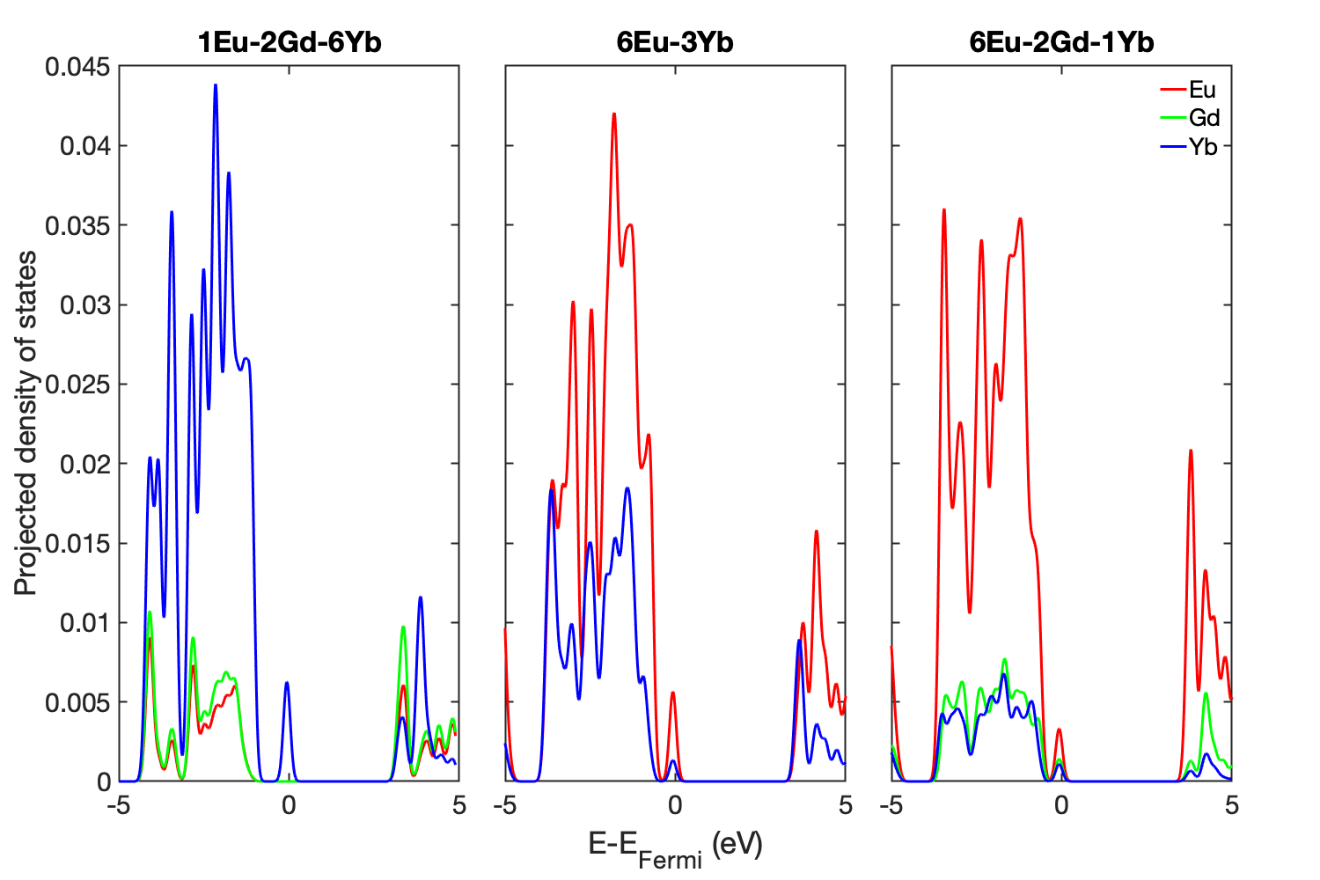


**Supplementary Figure 11**. Calculated rare earth projected density of states for three heterometallic clusters 6Yb-2Gd-1Eu (left), 6Eu-3Yb (center), and 6Eu-2Gd-1Yb (right). The PDOS identify the relative electron density localized on each of the three RE elements Eu (red), Yb (blue), and Gd (green).

**
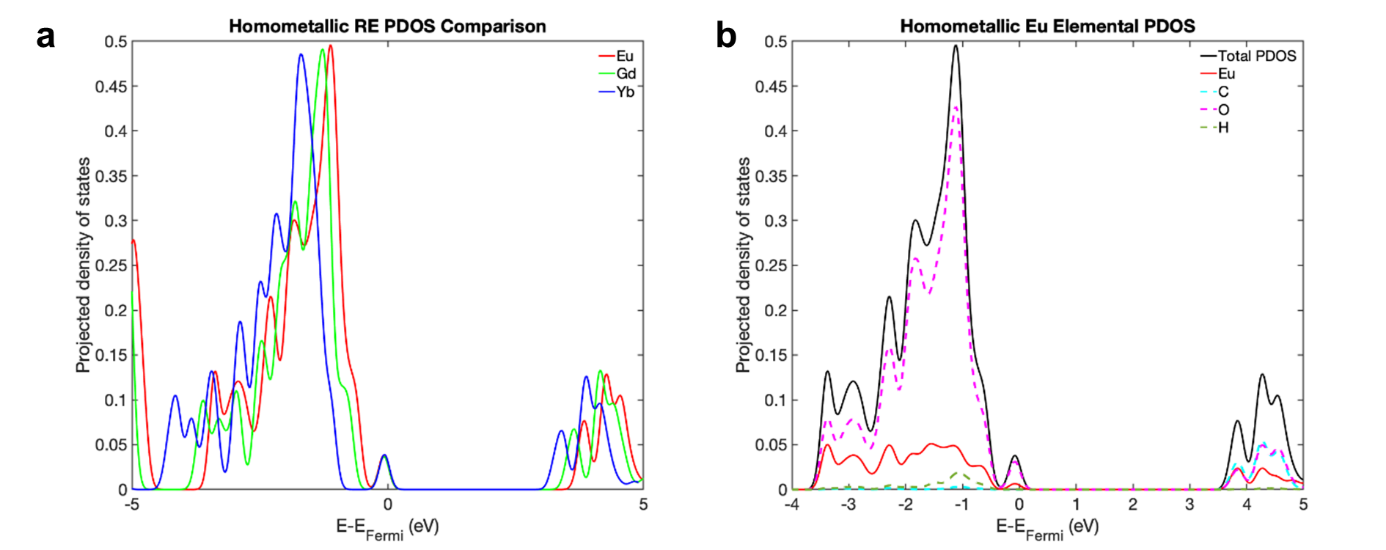
**

**Supplementary Figure 12**. a. Calculated RE PDOS for homometallic Eu (red), Gd (green), and Yb (blue) clusters plotted together for comparison. b. Full elemental PDOS for homometallic Eu cluster. The total elemental contributions to the PDOS (solild black) are identified for Eu (solid red), C (dashed cyan), O (dashed magenta), and H (dashed green). The homometallic Eu cluster was chosen as a representative examplar for the series of modeled clusters.


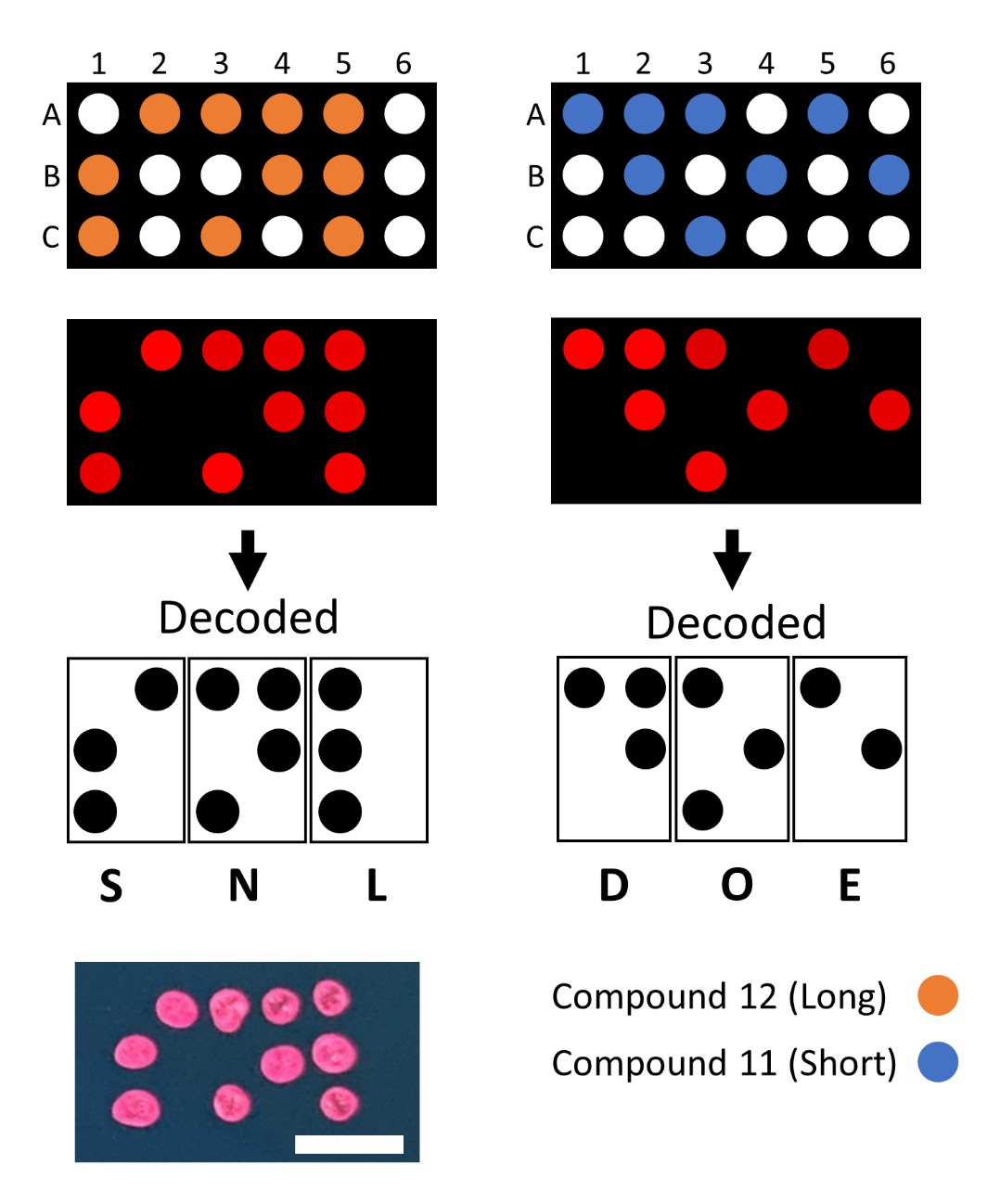


**Supplementary Figure 13.** Representation of an encoded message utilizing the braille alphabet in a 96 well plate, with the encoding based solely on the static luminescence of each compound. The intensity of each red dot is based on experimental data. Bottom image shows a photo of the SNL pattern drawn on glass using a PVA based ink under a black light. Scale bar = 5 mm.


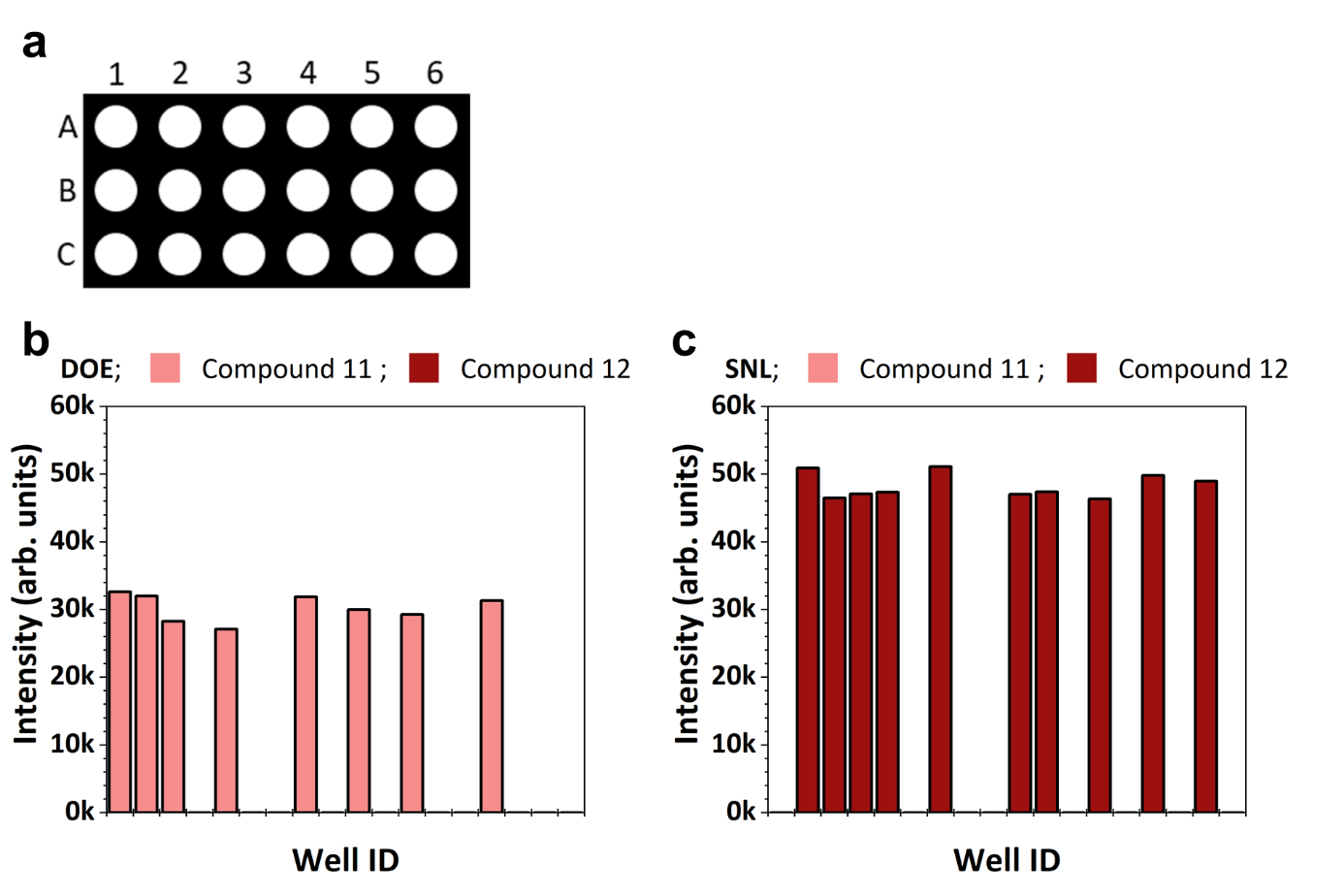


**Supplementary Figure 14.** Raw data from the single reads of SNL and DOE. Wellplate diagram included for clarity.

**Supplementary Table 2.** Raw data read from the static wellplate encoding shown in Supplementary Figure 7. Intensities given in arbitrary units. λ_ex_= 340 nm, λ_em_= 614 nm.

| SNL | | | | | | |
| --- | --- | --- | --- | --- | --- | --- |
|  | **1** | **2** | **3** | **4** | **5** | **6** |
| A | 19 | 50899 | 46509 | 47066 | 47345 | 20 |
| B | 51112 | 16 | 18 | 47037 | 47372 | 16 |
| C | 46353 | 17 | 49816 | 20 | 48981 | 18 |
| DOE | | | | | | |
|  | **1** | **2** | **3** | **4** | **5** | **6** |
| A | 32643 | 32006 | 28246 | 17 | 27110 | 17 |
| B | 16 | 31914 | 16 | 30012 | 20 | 29263 |
| C | 14 | 17 | 31326 | 15 | 16 | 16 |


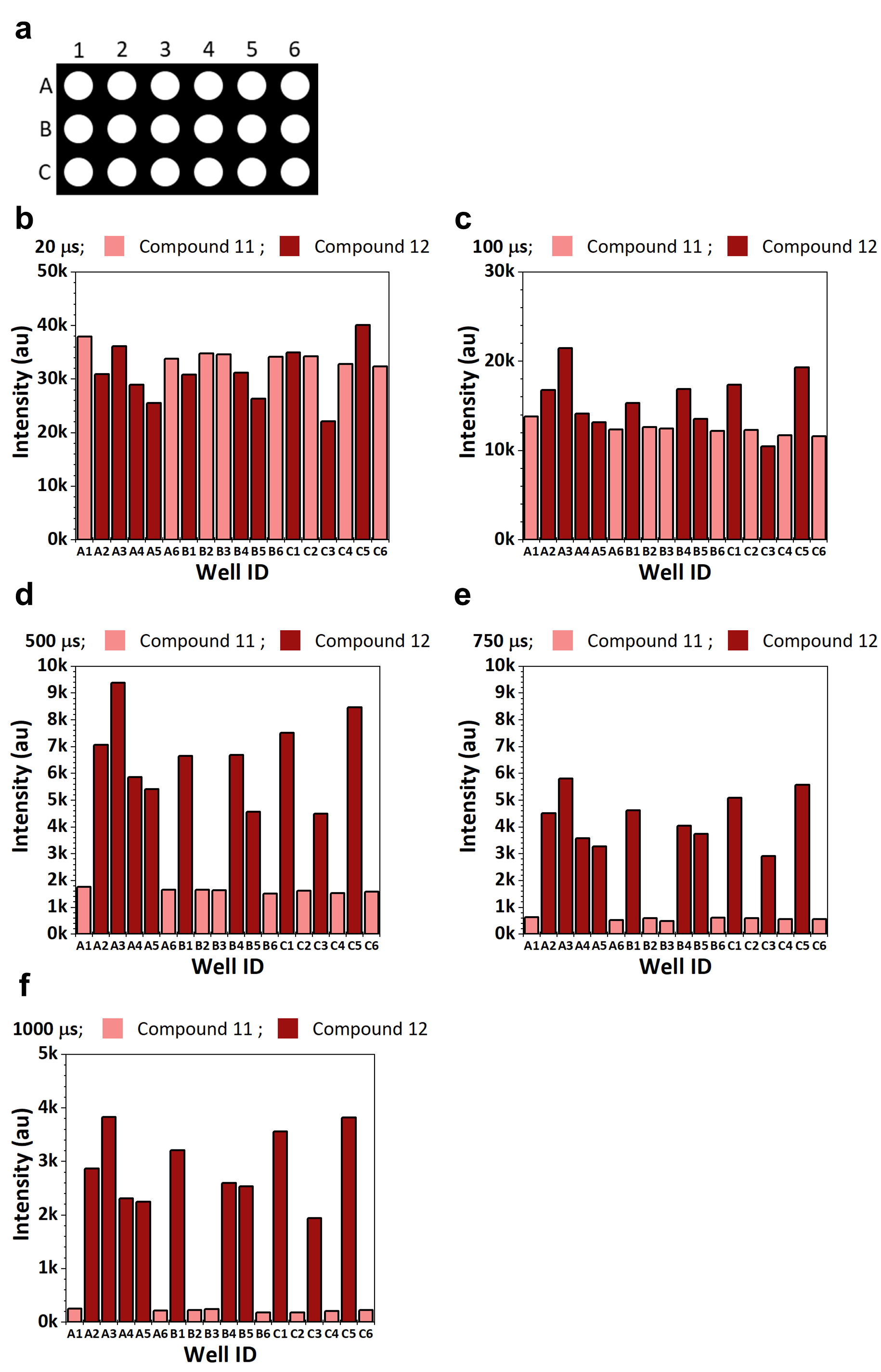


**Supplementary Figure 15.** a. Wellplate diagram for clarity. b-f. Histograms showing raw data for the dynamic encoding demonstrated in the main text, illustrating the changing relative intensities of each well over time.

**Supplementary Table 3.** Raw data read from the dynamic wellplate encoding shown in Supplementary Figure 8. Intensities given in arbitrary units. λ_ex_= 340 nm, λ_em_= 614 nm.

| **Static** | | | | | | |
| --- | --- | --- | --- | --- | --- | --- |
|  | **1** | **2** | **3** | **4** | **5** | **6** |
| **A** | 30055 | 36249 | 40156 | 35277 | 27724 | 26186 |
| **B** | 28177 | 28192 | 27144 | 33946 | 29383 | 27014 |
| **C** | 33396 | 26969 | 27257 | 26401 | 38117 | 25725 |
| **20 µs** | | | | | | |
|  | **1** | **2** | **3** | **4** | **5** | **6** |
| **A** | 37915 | 30913 | 36148 | 28979 | 25524 | 33782 |
| **B** | 30865 | 34835 | 34606 | 31197 | 26306 | 34208 |
| **C** | 35013 | 34257 | 22134 | 32818 | 40062 | 32324 |
| **100 µs** | | | | | | |
|  | **1** | **2** | **3** | **4** | **5** | **6** |
| **A** | 13801 | 16800 | 21465 | 14153 | 13156 | 12382 |
| **B** | 15345 | 12638 | 12476 | 16889 | 13534 | 12177 |
| **C** | 17376 | 12303 | 10470 | 11698 | 19297 | 11619 |
| **500 µs** | | | | | | |
|  | **1** | **2** | **3** | **4** | **5** | **6** |
| **A** | 1757 | 7071 | 9377 | 5870 | 5420 | 1654 |
| **B** | 6649 | 1663 | 1642 | 6681 | 4571 | 1515 |
| **C** | 7514 | 1615 | 4492 | 1524 | 8472 | 1589 |
| **750 µs** | | | | | | |
|  | **1** | **2** | **3** | **4** | **5** | **6** |
| **A** | 631 | 4512 | 5803 | 3573 | 3266 | 525 |
| **B** | 4616 | 594 | 492 | 4041 | 3751 | 607 |
| **C** | 5088 | 593 | 2921 | 558 | 5569 | 556 |
| **1000 µs** | | | | | | |
|  | **1** | **2** | **3** | **4** | **5** | **6** |
| **A** | 254 | 2869 | 3834 | 2310 | 2247 | 219 |
| **B** | 3207 | 227 | 244 | 2598 | 2536 | 185 |
| **C** | 3559 | 185 | 1942 | 206 | 3825 | 231 |


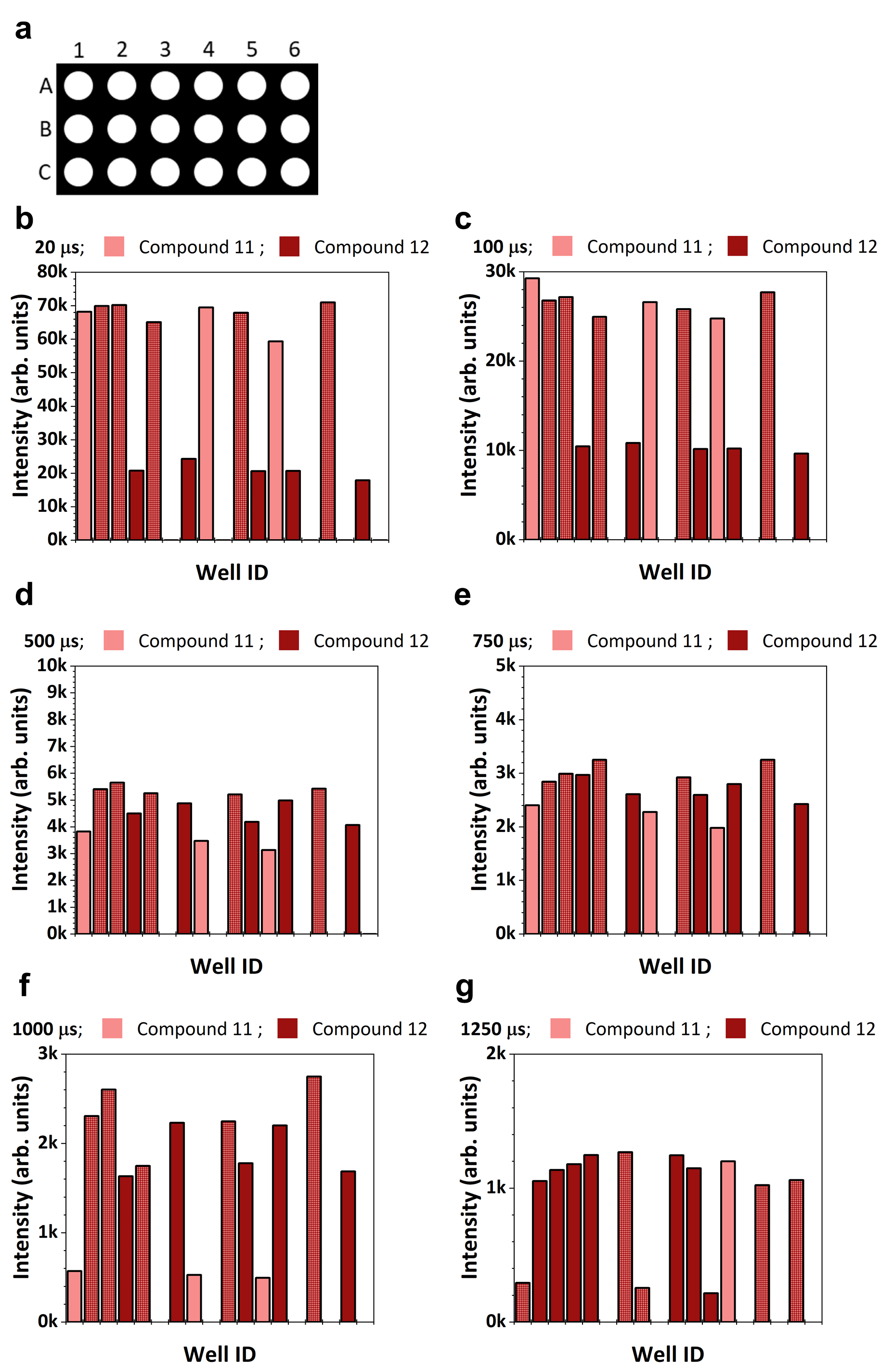


**Supplementary Figure 16.** a. Wellplate diagram for clarity. b-g. Histograms showing raw data for the double encoding demonstrated in the main text, illustrating the changing relative intensities of each well over time.

**Supplementary Table 4.** Raw data read from the double wellplate encoding shown in Supplementary Figure 9. Intensities given in arbitrary units. λ_ex_= 340 nm, λ_em_= 614 nm.

| **Static** | | | | | | |
| --- | --- | --- | --- | --- | --- | --- |
|  | **1** | **2** | **3** | **4** | **5** | **6** |
| **A** | 37734 | 43481 | 45443 | 10262 | 43892 | 17 |
| **B** | 10557 | 39126 | 15 | 44217 | 10500 | 34522 |
| **C** | 10013 | 14 | 46965 | 20 | 9010 | 17 |
| **20 µs** | | | | | | |
|  | **1** | **2** | **3** | **4** | **5** | **6** |
| **A** | 68193 | 69929 | 70193 | 20744 | 65102 | 31 |
| **B** | 24324 | 69492 | 10 | 67931 | 20657 | 59342 |
| **C** | 20692 | 14 | 71034 | 11 | 17892 | 32 |
| **100 µs** | | | | | | |
|  | **1** | **2** | **3** | **4** | **5** | **6** |
| **A** | 29262 | 26793 | 27159 | 10456 | 24961 | 0 |
| **B** | 10832 | 26594 | 0 | 25823 | 10165 | 24769 |
| **C** | 10212 | 0 | 27703 | 0 | 9645 | 0 |
| **500 µs** | | | | | | |
|  | **1** | **2** | **3** | **4** | **5** | **6** |
| **A** | 3825 | 5411 | 5650 | 4500 | 5256 | 0 |
| **B** | 4878 | 3477 | 0 | 5208 | 4191 | 3133 |
| **C** | 4987 | 0 | 5423 | 0 | 4070 | 1 |
| **750 µs** | | | | | | |
|  | **1** | **2** | **3** | **4** | **5** | **6** |
| **A** | 2403 | 2845 | 2992 | 2968 | 3252 | 0 |
| **B** | 2608 | 2278 | 0 | 2923 | 2597 | 1983 |
| **C** | 2798 | 0 | 3254 | 0 | 2424 | 0 |
| **1000 µs** | | | | | | |
|  | **1** | **2** | **3** | **4** | **5** | **6** |
| **A** | 572 | 2307 | 2605 | 1633 | 1749 | 0 |
| **B** | 2231 | 527 | 0 | 2247 | 1780 | 495 |
| **C** | 2202 | 0 | 2750 | 0 | 1687 | 0 |
| **1250 µs** | | | | | | |
|  | **1** | **2** | **3** | **4** | **5** | **6** |
| **A** | 572 | 2307 | 2605 | 1633 | 1749 | 0 |
| **B** | 2231 | 527 | 0 | 2247 | 1780 | 495 |
| **C** | 2202 | 0 | 2750 | 0 | 1687 | 0 |


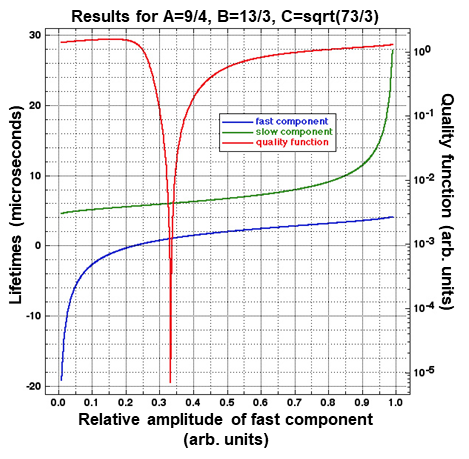


**Supplementary Figure 17.** An example of how the decay parameters can be recovered from the experimental parameters.


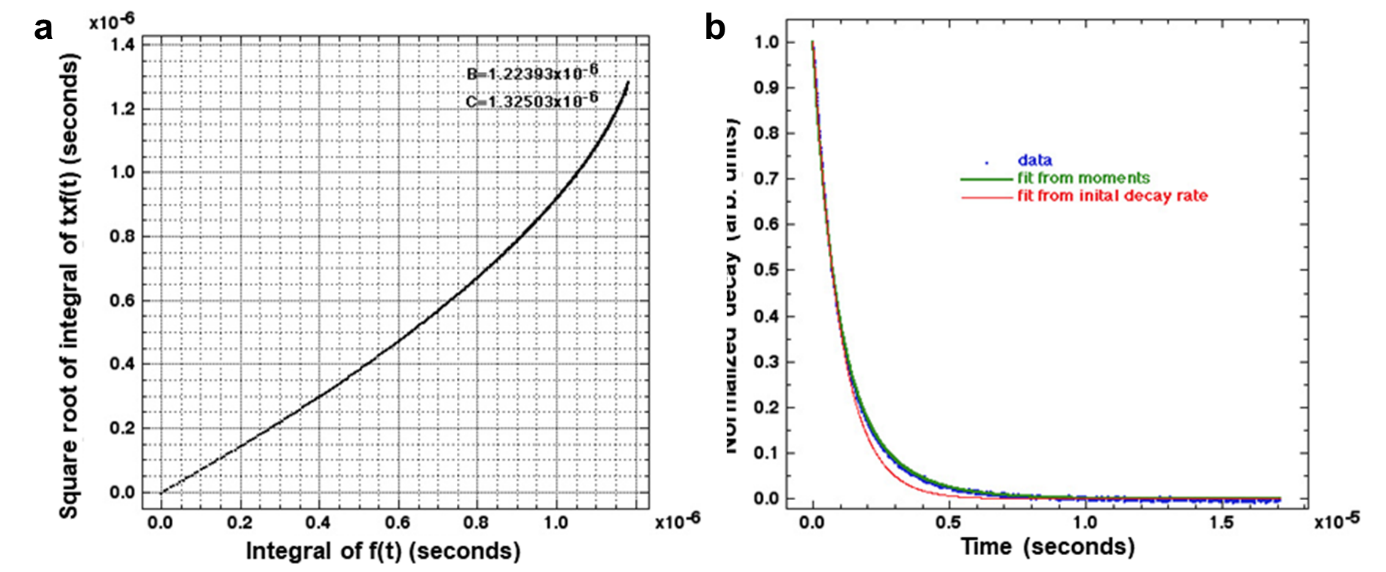


**Supplementary Figure 18.** a. A parametric plot of the partial integral of the second moment versus the partial integral of the first moment. b. The double exponential curve we obtained from the moments method (green) lies on top of the experimental data (blue). The red line has the same initial decay rate as the data, but fits poorly at larger times, simply because the data cannot be described by a single exponential decay.

**Supplementary Table 5.** The absolute quantum yield (QY) determined for compound **1** and **13**.

| Sample | 340 nm | 394 nm |
| --- | --- | --- |
| Compound 1 | 5.5% | 14.4% |
| Compound 13 | 1.1% | NA |

**Supplementary Discussion**

In the 6Yb-3Eu model, the first peak shows a combination of Yb and Eu contribution with the ratio of localized density on Eu:Yb as 2:1 while the second peak shows an inverted relationship. The proposed relaxation mechanisms, **Figure 3b**, indicate that fast decay rates in the heterometallic clusters are attributed to relaxation on the Yb atoms, which shows a higher localized density at the second peak. This second peak is at a higher energy and is closer to the initially excited energy states contributed by the organic linkers which would require less energy dissipation to transfer the charge to Yb prior to optical emission. Similar peak ordering is seen in the PDOS of the other fast decay rate compound, **11**. In the calculated PDOS, Figure **Supplementary Figure 11**, the second peak is dominated by Yb while the first peak has contributions from Gd>Eu>Yb even though the composition is dominated by Yb.

For the intermediate decay rate, the equimolar 3Eu-3Gd-3Yb cluster, **Figure 5b**, increasing Eu and Gd concentrations modifies the relative amplitudes of electron localization at the first and second peaks. The first peak becomes delocalized onto all RE elements, heavily localizing onto Eu. The resulting relative ratios for the first peak are not the equimolar ratio of the cluster, highlighting a strong preference for Eu at the conduction band edge. It also indicates that Gd, while optically inactive, reduces the density localized on the Yb species.

The second peak of the equimolar cluster shows an electron density localization that nearly maintains the equimolar ratio of 3:3:3 for Eu:Yb:Gd with Gd being the least of the three RE species. This distribution of localized density demonstrates that both Eu and Yb are still participating in possible high energy states that may receive charge from the excited organic linkers. This relative participation hypothesis agrees with the observed relative photoluminescence lifetimes comparing the equimolar cluster to the 3Eu-6Yb cluster.

The second intermediate decay rate compound, **2**, has a composition of 6Eu-3Yb. The calculated PDOS for compound 2, **Supplementary Figure 11**, shows a near equivalent mixing of Eu and Yb at the first peak. This is unique in that for all other modeled systems the Eu dominates the first peak at the conduction band edge. However, the second peak is dominated by Eu at a rate of 2:1 for Eu:Yb.

The composition of the slowest decay rate, compound **12**, has a composition heavily dominated by Gd: 1Eu-7Gd-1Yb. The Gd expectedly dominates the electron density localization as it is the primary metal in the cluster. However, for the two optically active elements, Eu and Yb, the Eu is the stronger participant in the conduction band structure and would be expected to dominate any optically active relaxation pathways. While at the second peak the Yb does show an increase peak amplitude compared to Eu, the long lifetime is expected as the Eu localizes more charge than Yb and there is substantial physical separation between the Eu and Yb elements in a Gd dominated cluster. The PDOS of the last slow decay rate compound, **10**, shows very minimal participation in the conduction band electronic structure by Yb, **Supplementary Figure 11**. This is expected as there is only one Yb atom in the 6Eu-2Gd-1Yb cluster. At both the first and second conduction band peaks Eu dominates and Gd shows more participation that Yb. The small concentration of Yb does not have enough interaction with the Eu atoms or organic linker excited states to be optically active.

A plot of the decay times as functions of the amplitude is shown in **Supplementary Figure 17.** The data are computed for discrete values of the amplitude, but the zero of the quality function is clearly at 1/3, and at this value the decay times are indeed 1 and 6. In this example we used the root for the negative sign in eq. 5. If we had used the positive sign the slow time becomes the fast time and vice versa, and thus $a$ and $1-a$ change accordingly, so either root is acceptable.

The full analysis of an experimental decay curve is also instructive. For this we have used a MOF that emits in the IR. A parametric plot of the integrals is shown in **Supplementary Figure 18** (left), from which $B=1.224\times{10}^{-6} s$ and $C=1.325\times{10}^{-6} s$ are obtained. The parameter $A=1.00\times{10}^{-6} s$ is obtained from the initial slope of the decay. Using the method described in the main text gives $a=0.610, \tau_{1}=0.777\times{10}^{-6} \mathrm{and} \tau_{2}=1.813\times{10}^{-6} s.$ **Supplementary Figure 18** (right) shows the quality of the fit of this double exponential decay to the data. The fit lies perfectly on top of the experimental data. Also shown (red line) is the single exponential decay that has the same initial decay rate as the experimental data. This function departs significantly from the experimental data, which is an indication of the need for a double exponential fit.
